# Prevalence of respiratory viruses among adults, by season, age, respiratory tract region and type of medical unit in Paris, France, from 2011 to 2016

**DOI:** 10.1371/journal.pone.0180888

**Published:** 2017-07-14

**Authors:** Benoit Visseaux, Charles Burdet, Guillaume Voiriot, François-Xavier Lescure, Taous Chougar, Olivier Brugière, Bruno Crestani, Enrique Casalino, Charlotte Charpentier, Diane Descamps, Jean-François Timsit, Yazdan Yazdanpanah, Nadhira Houhou-Fidouh

**Affiliations:** 1 IAME, UMR 1137, INSERM, Université Paris Diderot, Sorbonne Paris Cité, Laboratoire de Virologie, Hôpital Bichat, AP-HP, Paris, France; 2 IAME, UMR 1137, INSERM, Université Paris Diderot, Sorbonne Paris Cité, Services des Maladies Infectieuses et Tropicales, Hôpital Bichat, AP-HP, Paris, France; 3 IAME, UMR 1137, INSERM, Université Paris Diderot, Sorbonne Paris Cité, Réanimation Médicale et Infectieuses, Hôpital Bichat, AP-HP, Paris, France; 4 Service de Pneumologie B, Hôpital Bichat, AP-HP, Paris, France; 5 Service de Pneumologie A, Hôpital Bichat, AP-HP, Paris, France; 6 Service d’Accueil des Urgences, Hôpital Bichat, AP-HP, Paris, France; Public Health Agency of Canada, CANADA

## Abstract

**Background:**

Multiplex PCR tests have improved our understanding of respiratory viruses’ epidemiology by allowing their wide range detection. We describe here the burden of these viruses in hospital settings over a five-year period.

**Methods:**

All respiratory samples from adult patients (>20 years old) tested by multiplex-PCR at the request of physicians, from May 1 2011 to April 30 2016, were included retrospectively. Viral findings are reported by season, patient age group, respiratory tract region (upper or lower) and type of clinical unit (intensive care unit, pneumology unit, lung transplantation unit and other medical units).

**Results:**

In total, 7196 samples (4958 patients) were included; 29.2% tested positive, with viral co-infections detected in 1.6% of samples. Overall, two viral groups accounted for 60.2% of all viruses identified: picornaviruses (rhinovirus or enterovirus, 34.3%) and influenza (26.6%). Influenza viruses constituted the group most frequently identified in winter (34.4%), in the upper respiratory tract (32%) and in patients over the age of 70 years (36.4%). Picornavirus was the second most frequently identified viral group in these populations and in all other groups, including lower respiratory tract infections (41.3%) or patients in intensive care units (37.6%).

**Conclusion:**

This study, the largest to date in Europe, provides a broad picture of the distribution of viruses over seasons, age groups, types of clinical unit and respiratory tract regions in the hospital setting. It highlights the burden associated with the neglected picornavirus group. These data have important implications for the future development of vaccines and antiviral drugs.

## Introduction

Interest in respiratory viruses has increased recently, due to the identification of several new viruses [1] and the threat posed by others able to cross the interspecies barriers, as for severe respiratory acute syndrome (SRAS) [2], avian influenza A H5N1, H7N9, H1N1v2009 [3,4] and MersCoV [5].

Before the introduction of molecular approaches, the mainstays in the diagnosis of viral respiratory tract infections were serology, virus isolation in cell culture and immunofluorescence assays. All these techniques have a low sensitivity or can detect only a limited number of viruses. Sensitivity and the time required to obtain a result have been improved by the recent development of molecular tests, which can also detect viruses that were previously undetected, such as coronaviruses NL63, HKU1 and bocaviruses. The use of such tests has also demonstrated that the carriage of respiratory viruses is very rare in adults (2.1%) [6].

The recent development of new multiplex PCR tests (mPCR) has facilitate the rapid detection of a broad range of respiratory viruses in clinical specimens and are increasingly used [7–10]. However, such tests are expensive and not yet taken into account by regulations or guidelines, so they are widely used. Nevertheless, their use has already improved descriptions of the epidemiology of respiratory viruses in various high-risk populations, such as neonates and preterm infants [11,12], children [13,14], immunocompromised patients [15], and the elderly [16]. For the general adult population, recent studies have shown that viral pathogens can be detected in 53% of patients attending the emergency department with acute respiratory symptoms [17], and in 23 to 28% of those with community-acquired pneumonia [18,19]. In the ICU, respiratory viruses are detected in 30 to 36% of patients with community-acquired pneumonia or nosocomial pneumonia, and are associated with high mortality rates [20–23]. However, the studies published to date were subject to several limitations, as they were based on different methods for detecting respiratory viruses, and were performed in specific populations, often only during the winter.

The objective of this study was to retrospectively describe the epidemiology of respiratory viruses with the use of mPCR in adults hospitalized in France, over a five-year period. Results are presented by season, type of clinical unit, patient age group and respiratory tract region involved.

## Methods

### Patients and period

All respiratory samples from adult patients (>20 years old) tested for respiratory viruses at the request of a physician from May 1, 2011, to April 30, 2016, were included in this study retrospectively. Our hospital group encompasses five hospitals (Beaujon, Bichat-Claude Bernard, Louis Mourier, Bretonneau and Adelaïde Hautval), all located in the Paris area. Three have intensive care units, emergency, infectious diseases, internal and general medicine departments, and the other two are geriatric hospitals. All nasopharyngeal swabs, bronchial aspirates and bronchoalveolar lavages were placed in Virocult MWE (Sigma, St Louis, MO, USA) transport medium for transport to the virology laboratory of Bichat-Claude Bernard Hospital. Repeat samples, defined as samples taken from the same part of the respiratory tract (upper or lower) of the same patient in the same month, were discarded.

In accordance with French ethical rules for epidemiological surveillance studies and the rules in place at APHP (*Assistance Publique—Hôpitaux de Paris*) we obtained the non-opposition of the patient at hospital admission.

### Multiplex PCR tests

Several different mPCR tests were used during the study period: the Respifinder^®^ 19 (Pathofinder^®^, Maastricht, the Netherlands) from May 2011 to February 2012, Respifinder^®^ 22 (Pathofinder^®^, Maastricht, the Netherlands) from March 2012 to December 2016 and Anyplex^™^ II RV16 (Seegene^®^, Seoul, South Korea) from January to April 2014. From June 2012 to April 2016, the Filmarray Respiratory Panel (BioFire Diagnostics, Salt Lake City, USA) was also used in response to specific requests for rapid diagnosis from the physicians of intensive care units or pneumology units. These changes in the tests used over time were made to decrease the time to results. None of these tests could differentiate between rhinovirus and enterovirus. These two types of virus were therefore grouped together as “picornavirus” (rhinovirus + enterovirus) for the purposes of this analysis. The Respifinder^®^ 19 and FilmArray^™^ tests, accounting for 26.4% of all the tests included in the analysis, cannot detect bocaviruses. The various tests used have been reported to have similar performances [8,9,24], as confirmed by our internal comparisons and method validations. Their reliability was also assessed throughout the study period by QCMD controls (Glasgow, UK).

### Statistical analysis

We compared the distribution of viral findings by (i) month of sampling, (ii) area of the respiratory tract sampled (upper or lower), (iii) type of medical unit and (iv) patient age. The sampling areas were defined as upper respiratory tract (URT) for all nasopharyngeal swabs and aspirates, and lower respiratory tract (LRT) for all bronchial aspirates and bronchoalveolar lavages. Four groups of prescribing units were defined according to the section of hospitals: intensive care units (ICU), pneumology units, lung transplantation units and other units. The “other” units were mostly the emergency department and infectious diseases, internal medicine, nephrology, and geriatrics units. Patients were stratified into several age groups: 20–40 years, 41–50 years, 51–60 years, 61–70 years and >70 years. χ^2^ tests and Fisher’s exact tests, performed with R software v3.2.0, were used for comparisons between groups, as appropriate. All tests were performed with a type I error of 0.05.

## Results

During the study period, 7196 samples from 4958 patients were included in the analysis. In accordance with our definition and exclusion of repeat samples, no sample obtained, within 30 days, from the same area of the respiratory tract of a given patient as the initial sample was included. The median interval between two included samples from the same respiratory tract area of the same patient was 133 days (interquartile range [IQR] = 63–223). Median patient age was 59.9 years [IQR = 48.0–70.0] and 40.0% [IC95 = 38.2–41.4%] of the patients were female. Overall, 2098 (29.2%, [IC95 = 28.1–30.2]) samples tested positive for a respiratory virus and there were 118 viral co-infections (1.6%, [IC95 = 1.3–2.0%]). The groups of viruses identified were, in descending order of prevalence: picornavirus (n = 762, 34.3% [IC95 = 32.2–36.3%]), influenza (n = 592, 26.6% [IC95 = 24.8–28.5%]), coronavirus (n = 260, 11.7% [IC95 = 10.4–13.1%]), respiratory syncytial virus (RSV) (n = 215, 9.7% [IC95 = 8.5–11.0%]), parainfluenza (n = 179, 8.1% [IC95 = 7.0–9.3%]), metapneumovirus (n = 126, 5.7% IC95 = 4.7–6.7%]), adenovirus (n = 61, 2.7% [IC95 = 2.1–3.5%]) and bocavirus (n = 28, 1.3% [IC95 = 0.8–1.8%]).

**Fig 1:**
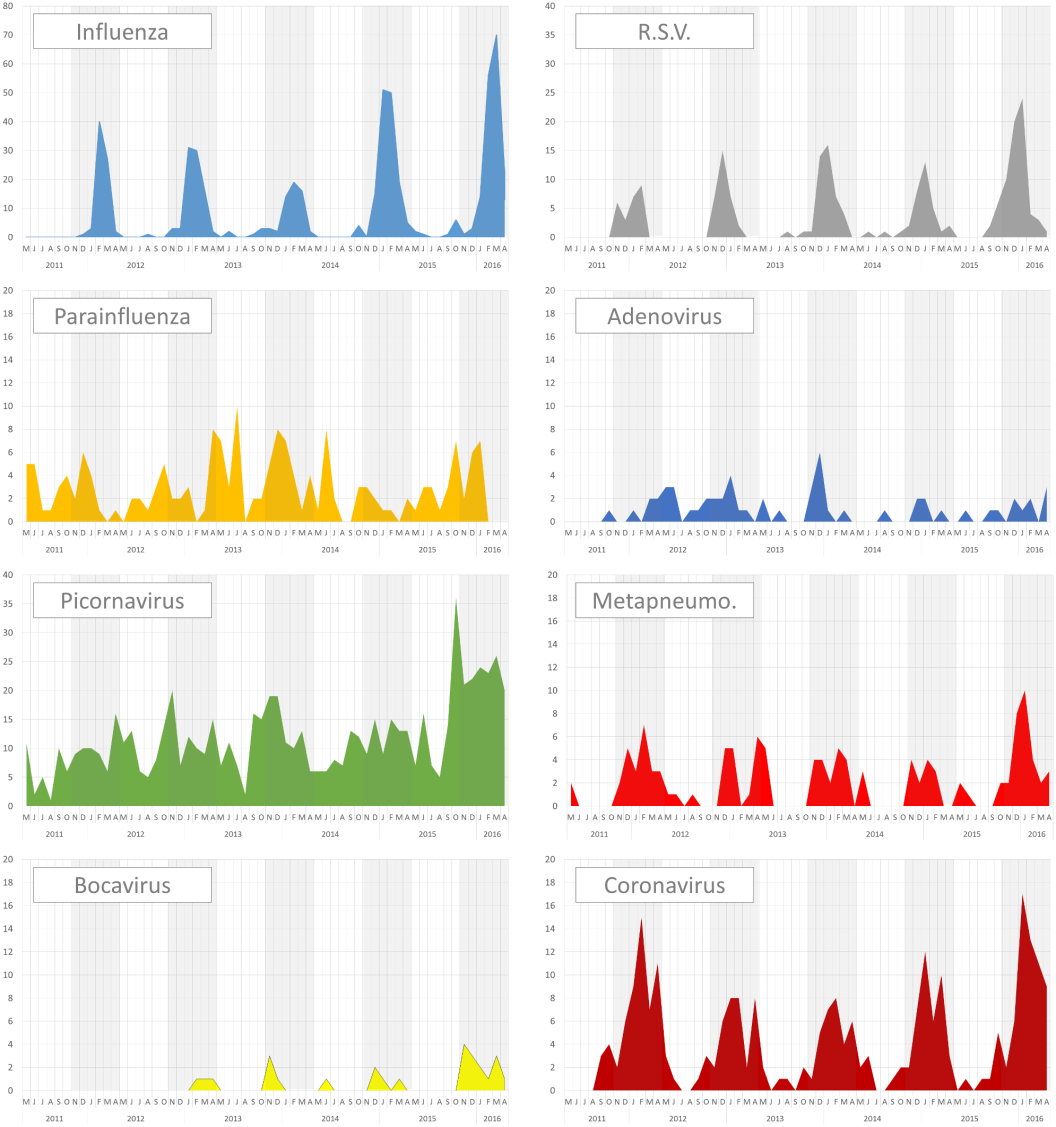
Seasonality of respiratory viruses. For each group of viruses, the monthly number of identifications is shown for the entire study period.

The overall distribution of viruses, from year to year, is depicted in Fig 1 and S1 Fig The variation between years was driven mostly by influenza epidemic variations and by a specific variation in the summer of 2012. The number of tests performed annually increased steadily over the study period, from 634 to 1640 samples (Table 1).

**Table 1:** Number of samples tested, positivity rates and viral findings by season, area of the respiratory tract sampled, type of medical unit and age group.

	Seasonality		Respirtory tract area		Medical unit				Age strata					
	Winter	Summer	URT	LRT	I.C.U.	Medicine	Pneumology	Lung transplant	20–30	31–40	41–50	51–60	61–70	>80
Number of samples	4621	2151	3865	2729	1944	2464	778	1586	366	584	971	1550	1745	704
Positivity rates (%) [IC95]	33.3 [32.2–34.3]	22.2 [20.9–23.5]	32.6 [31.1–34.1]	26.2 [24.6–27.9]	24.6 [22.7–26.6]	31.6 [29.8–33.5]	30.8 [27.6–34.2]	34.2 [31.9–36.6]	32.8 [28.0–37.9]	32.5 [28.7–36.5]	29.1 [26.3–32.1]	29.9 [27.6–32.2]	29.1 [26.9–31.2]	32.4 [28.9–36.0]
Identified viruses (n, %)														
*Influenza*	520 (32.6%)	21 (4.3%)	413 (31.9%)	117 (16.0%)	122 (25.1%)	313 (39.4%)	23 (11.3%)	67 (11.4%)	42 (35.6%)	51 (26.7%)	64 (21.8%)	89 (18.1%)	140 (26.6%)	93 (40.6%)
*RSV*	191 (12.0%)	13 (2.7%)	123 (9.5%)	75 (10.2%)	48 (9.9%)	67 (8.4%)	13 (6.4%)	72 (12.3%)	5 (4.2%)	12 (6.3%)	23 (7.8%)	51 (10.4%)	55 (10.4%)	27 (11.8%)
*Parainfluenza*	83 (5.2%)	88 (18.2%)	79 (6.1%)	89 (12.1%)	37 (7.6%)	35 (4.4%)	18 (8.9%)	84 (14.3%)	5 (4.2%)	14 (7.3%)	27 (9.2%)	49 (10.0%)	52 (9.9%)	8 (3.5%)
*Adenovirus*	39 (2.4%)	18 (3.7%)	30 (2.3%)	24 (3.3%)	11 (2.3%)	23 (2.9%)	7 (3.4%)	13 (2.2%)	7 (5.9%)	13 (6.8%)	12 (4.1%)	13 (2.6%)	9 (1.7%)	0 (0%)
*Picornaviridae*	421 (26.4%)	287 (59.4%)	388 (30.0%)	302 (41.2%)	183 (37.6%)	218 (27.5%)	89 (43.8%)	238 (40.5%)	45 (38.1%)	69 (36.1%)	118 (40.1%)	184 (37.4%)	182 (34.5%)	46 (20.1%)
*Metapneumovirus*	101 (6.3%)	18 (3.7%)	69 (5.3%)	47 (5.3%)	34 (7%)	37 (4.7%)	15 (7.4%)	32 (5.5%)	2 (1.7%)	6 (3.1%)	13 (4.4%)	37 (7.5%)	17 (3.2%)	23 (10.0%)
*Bocavirus*	25 (1.6%)	1 (0.2%)	16 (1.2%)	10 (1.2%)	5 (1.0%)	9 (1.1%)	0 (0%)	12 (2.0%)	3 (2.5%)	3 (1.6%)	3 (1.0%)	6 (1.2%)	8 (1.5%)	3 (1.3%)
*Coronavirus*	213 (13.4%)	37 (7.7%)	175 (13.5%)	69 (9.4%)	47 (9.7%)	92 (11.7%)	38 (18.7%)	69 (11.8%)	9 (7.6%)	23 (12.0%)	34 (11.6%)	63 (12.8%)	64 (12.1%)	29 (12.7%)

### Seasonality

**Fig 2:**
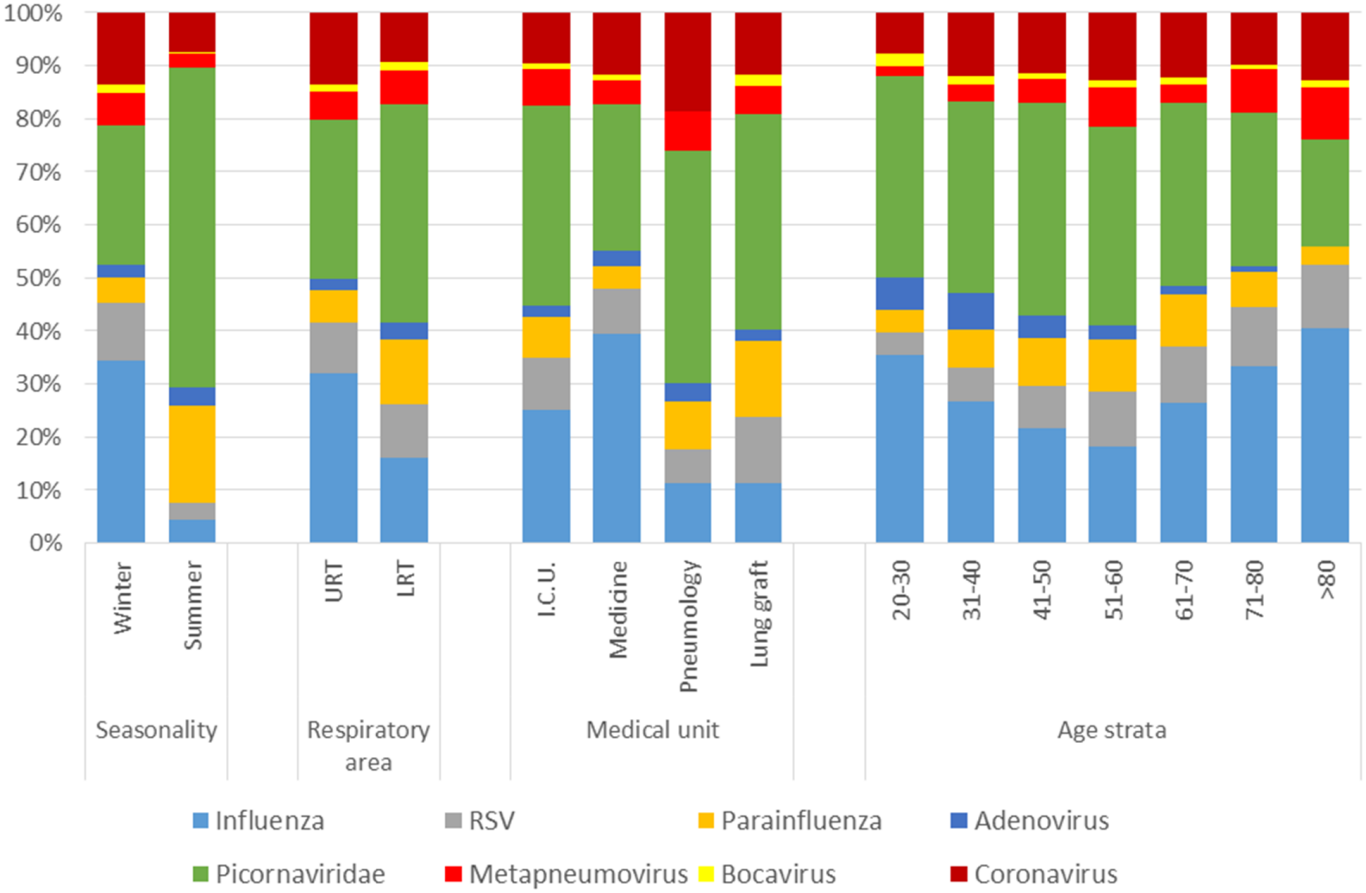
Distribution of viruses by season, part of the respiratory tract sampled, type of medical unit and age group.

The monthly cumulative results and viral distribution across the study period are depicted in Table 2 and Fig 1. The cumulative numbers of samples and the frequency of positive tests were higher from November to April, a period extending from late fall to early spring. We refer to this period as the “winter period” here. The frequency of positive tests for viruses was higher during the winter period than during the summer period (33.3 vs. 22.2%, *p*<0.0001), and, as expected, the distribution of viruses differed between these two periods (Fig 2, *p*<0.0001). The distribution of influenza followed the typical seasonal pattern observed in Europe, with active circulation from January to March. RSV, coronavirus, metapneumovirus and bocavirus were also more frequently detected in the winter, with a period of circulation extending from November to May. Two groups of viruses circulated throughout the year: picornavirus and parainfluenza virus. Together, these two groups accounted for 78.6% of all viruses detected during the summer period and picornaviruses constituted the second most frequent group of viruses during the winter (26.1%), after influenza (34.4%). Adenovirus had a more complex seasonal pattern over the years covered by this study. It was very rare in adults during 2011 and 2014, circulated throughout the year in 2012 and caused an epidemic exclusively during the winter in 2013.

**Table 2:** Monthly cumulative number of samples and seasonal distribution of the respiratory viruses detected by multiplex PCR over the five-year study period.

	January*	February*	March*	April*	May	June	July	August	September	October	November*	December*
Total number of samples	962	897	#	665	364	#	334	218	373	485	#	677
Total number of positive samples	349	361	#	168	79	#	53	33	85	142	#	239
Positivity rate												
Mean	36.30%	40.20%	#	25.30%	21.70%	#	15.90%	15.10%	22.80%	29.30%	#	35.30%
(min—max)	(32.3–40.4%)	(33.8–50.7%)	(20.4–38.7%)	(20.5–39.5%)	(14.5–36.4%)	(13.7–30.2%)	(10.8–22.4%)	(9.8–28.1%)	(18.9–30.0%)	(23.1–40.4%)	(18.8–41.8%)	(30.3–40.6%)
Viruses identified:												
Influenza	113 (31.7%)	195 (52.8%)	148 (53.0%)	33 (18.6%)	2 (2.4%)	3 (3.5%)	0 (0%)	1 (3.2%)	2 (2.4%)	13 (8.8%)	7 (4.4%)	24 (9.5%)
RSV	67 (18.8%)	27 (7.3%)	8 (2.9%)	3 (1.7%)	0 (0%)	1 (1.2%)	0 (0%)	2 (6.5%)	2 (2.4%)	8 (5.4%)	26 (16.5%)	60 (23.7%)
Parainfluenza	22 (6.2%)	6 (1.6%)	2 (0.7%)	15 (8.5%)	14 (16.9%)	21 (24.7%)	18 (34%)	3 (9.7%)	11 (13.1%)	21 (14.3%)	14 (8.9%)	24 (9.5%)
Adenovirus	9 (2.5%)	3 (0.8%)	5 (1.8%)	5 (2.8%)	5 (6.0%)	4 (4.7%)	1 (1.9%)	2 (6.5%)	2 (2.4%)	4 (2.7%)	5 (3.2%)	12 (4.7%)
Picornavirus	66 (18.5%)	67 (18.2%)	67 (24.0%)	70 (39.5%)	42 (50.6%)	48 (56.5%)	33 (62.3%)	20 (64.5%)	61 (72.6%)	83 (56.5%)	78 (49.4%)	73 (28.9%)
Metapneumo.	24 (6.7%)	19 (5.1%)	10 (3.6%)	12 (6.8%)	13 (15.7%)	2 (2.4%)	0 (0%)	1 (3.2%)	0 (0%)	2 (1.4%)	12 (7.6%)	24 (9.5%)
Bocavirus	3 (0.8%)	2 (0.5%)	5 (1.8%)	2 (1.1%)	0 (0%)	1 (1.2%)	0 (0%)	0 (0%)	0 (0%)	0 (0%)	7 (4.4%)	6 (2.4%)
Coronavirus	53 (14.8%)	50 (13.6%)	34 (12.2%)	37 (20.9%)	7 (8.4%)	5 (5.9%)	1 (1.9%)	2 (6.5%)	6 (7.1%)	16 (10.9%)	9 (5.7%)	30 (11.9%)

* Months associated with the winter period.

### Respiratory tract area

The area of the respiratory tract sampled was indicated for 6594 samples (91.6%): 58.6 of these samples came from the URT and 41.4% came from the LRT. The results are depicted in Fig 2 and Table 2.

The URT was more frequently sampled during the winter (77.5% vs. 55.5% for the LRT, *p*<0.0001) and the positivity rate was higher for the URT than for the LRT (32.0% [IC95 = 31.1–34.1] vs. 25.3% [20.9–23.5], p<0.0001). The distribution of viral groups differed significantly between the URT and LRT (*p*<0.0001). The three most frequent viral groups in URT were influenza (32.0%, [IC95 = 29.4–34.6]), picornavirus (30.0%, [27.5, 32.6]) and coronavirus (13.1%, [11.7–15.5]). The most frequent viral groups in the LRT were picornavirus (41.3%, [37.6–44.9]), parainfluenza (11.7%, [9.8–14.7]), influenza (16.3%, [13.4–18.8]) and RSV (10.5%, [8.1–12.7]).

### Medical units

The results obtained for the 6532 (90.7%) samples for which the type of medical unit was recorded are depicted in Fig 2 and Table 2. Positivity rates differed significantly between types of medical unit (*p*<0.001), ranging from 23.8 in ICUs to 34.0% in lung transplantation units. The distribution of viruses also differed between types of medical units (*p*<0.001): picornaviruses were the most frequent group in ICUs (37.6%, [IC95 = 33.2–42.0]), pneumology units (43.9%, [IC95 = 36.9–51.0]) and lung transplantation units (40.5%, [IC95 = 36.5–44.6]). By contrast, in other medical units, influenza and picornavirus accounted for 39.4% [IC95 = 36.0–42.9] and 27.4% [IC95 = 24.4–30.7] of all viruses, respectively. The proportion of samples obtained during the winter differed significantly between types of medical unit: 58.9% [56.5–61.4] for lung transplantation units, 65.1% [63.0–67.2] for ICUs, 72.3% [69.1–75.4] for pneumology units and 75.6% [74.0–77.4] for other medical units (p<0.001), consistent with the greater focus on virus identification during influenza epidemics.

Infections of the LRT area are more representative of severe infections and rates of sampling for this part of the respiratory tract are constant through the year. We therefore analyzed the viral distribution between types of medical unit separately for LRT samples (S2 Fig). For LRT samples, positivity rates were 20.6% [IC95 = 18.0–23.3] for ICUs, 24.0% [19.3–29.2] for pneumology units, 33.0% [30.0–36.1] for lung transplantation units and 21.5% [18.1–25.2] for other units. The prevalence of influenza was higher for ICUs than for all the other types of units (24.6% [18.9–31.2], versus 11.3% [8.8–14.3], *p*<0.001). The picornavirus group was the most frequent viral group in all units, at frequencies ranging from 41.4% [34.5-48-5] of all viruses detected in ICUs to 43.0% [34.0–52.3] in lung transplantation and the other medical units group.

### Ages

Positivity rates did not differ significantly between age groups, ranging from 27.2% to 31.8% (*p* = 0.61). However, the distribution of viruses differed between age groups (Fig 2 and Table 2, *p*<0.0001). Picornaviruses accounted for between 20.1% [IC95 = 15.1–25.9] and 40.1% [IC95 = 34.5–46.0] of the viruses identified in all age groups. This group of viruses was the most frequent in all but the most extreme age groups considered (20–30 and >80 years), in which influenza was the most frequent (35.6% [IC95 = 27.0–44.9] and 40.6% [IC95 = 34.2–47.3], respectively).

An analysis of URT and LRT samples separately (S3 Fig) showed influenza to be less frequent in the LRT than in the URT for all age groups. The picornavirus group was the group of viruses most frequently detected in LRT samples, for all age groups.

### Viral co-infections

Only 118 of the 7196 samples tested (1.6%, [IC95 = 1.3–2.0%]) corresponded to viral co-infections, six of which were co-infections involving three viruses. The proportion of viruses identified in co-infections differed between groups: 7.3% [IC95 = 5.3–9.7] for influenza, 9.4% [7.5, 11.8] for picornavirus, 10.6% [6.5–16.1] for parainfluenza, 11.9% [6.8–18.9] for metapneumovirus, 13.6% [9.3–18.9] for RSV, 15.8% [11.6–20.8] for coronavirus, 24.6% [14.5–37.3] for adenovirus and 28.6% [13.2–48.7] for bocavirus (*p<0*.*001*). All groups of viruses seemed to be able to exist alongside other viruses in co-infections, and no preferential association between respiratory viruses was identified.

## Discussion

The burden of respiratory viruses is well established in children. For example, we recently described the global epidemiology of respiratory viruses in children treated in our hospital group [25]. We found that the broad-range detection provided by multiplex PCR greatly improved the detection of respiratory viruses, and, thus our understanding of their epidemiology. We also found differences between children and adults, as also reported by other groups, especially for influenza [26]. Interest in respiratory viruses present in adult populations is also increasing. However, many previous studies have focused exclusively on the winter period or influenza detection and may have underestimated the potential role of other respiratory viruses. A Canadian study performed with Canada's national hospitalization database showed that influenza and other respiratory viruses are associated with morbidity requiring hospitalization, in both children and adult populations [27]. Other studies have also described the high frequency of non-influenza viruses in adults hospitalized in China [17,28], Spain [29] and France [18]. However, due to differences in populations, age groups, climate conditions, sampling or laboratory methods, the incidence and seasonality of respiratory viruses may differ slightly between countries and studies. Moreover, only limited data are available for the distribution of respiratory viruses in the lower respiratory tract.

Our retrospective study, one of the largest such studies in Europe, provides a broad snapshot of the epidemiology of respiratory viruses in hospital settings over a five-year period, based on the results of mPCR testing. In our study, 29.2% of the 7196 samples tested were positive for at least one respiratory virus. Nasopharyngeal samples, collected mostly during the winter period, had a positivity rate of 32.0%. This is slightly lower than reported in previous clinical studies based on mPCR testing of nasopharyngeal samples, in which positivity rates ranged from 40 to 45% [30–32]. This difference can be explained by the use of a strict case definition linked to the prospective aspects of these studies, whereas our study was retrospective and may be more representative of the real conditions in our hospital units. The lower respiratory tract was sampled at a constant frequency throughout the year and its positivity rate was non-negligible, at 26.2%. This finding is consistent with those of previous studies on pneumonia cases admitted to ICUs, in which positivity rates ranged from 16% to 36.4% [20,21,33]. Interestingly, the main viral group identified in the LRT samples was also the most neglected: the picornavirus group. This group of viruses was the leading group present in the LRT of patients from all age groups (from 27.6 to 56.3%) and types of medical units (>40% of all identified viruses).

The role of picornavirus in pneumonia is increasingly being studied. It is commonly thought that picornaviruses, which cause most “common colds”, are associated only rarely, if ever, with severe pneumonia, and that the detection of viruses from this group in cases of severe pneumonia must reflect asymptomatic carriage. However, despite the frequent detection of picornaviruses in asymptomatic children [34,35], recent studies have shown that the asymptomatic carriage of respiratory viruses, including picornaviruses, is rare in asymptomatic adults [6,19]. It has also been suggested that the viruses of this group are better adapted for URT infection than for LRT infection, as they replicate in vitro more effectively at 33°C than at 37°C [36]. However, experimental picornavirus infections are not restricted to the URT and are also detected in the LRT [37]. They may also be associated with increases in the number of the bacteria classically associated with secondary infections in the bacterial microbiota, accounting for the predisposition to bacterial otitis media, sinusitis and pneumonia conferred by picornavirus [38]. Picornaviruses are known to be associated with asthma exacerbation and chronic obstructive pulmonary disease, and they have also been implicated directly in the development of asthma [39]. Some previous studies have revealed an association between specific subtypes of picornaviruses and greater pathogenicity in immunocompromised patients [40,41]. More importantly, picornaviruses are increasingly being associated with both community- and hospital-acquired severe pneumonia [20–23,42]. In these studies, viral pneumonia was frequently found to be associated with picornaviruses and to display non-negligible pathogenicity or a complicated clinical course in co-infections involving both bacteria and viruses. The huge predominance of picornaviruses in the LRT observed in our study also highlights the potential role of these viruses in severe pneumonia.

The pathogenicity of other groups of viruses is also increasingly being described. Parainfluenza, identified as the second most frequent group of viruses in the LRT in our study, has also recently been associated with a mortality rate of 17% in immunocompromised patients [43] and with about 400–500 deaths per year in patients over the age of75 years in the Netherlands [16].

In this study, influenza was very rare in the highly vaccinated population of lung transplant recipients, but was highly prevalent in older patients. This finding highlights the need to increase vaccination rates in the elderly. Indeed, despite the objective of 75% vaccination rates in at-risk populations in France, only 60.2% of these populations in 2009 and 48.9% in 2013 were effectively vaccinated [44]. In our study, influenza was also highly prevalent in the URT, but not the LRT, of patients under the age of 40 years, suggesting that this population present less severe disease due to this virus but nevertheless play a major role in driving the influenza epidemic, as also suggested for populations of children in previous studies [45]. Not all our mPCR tests were able to fully discriminate influenza subtypes, but the data obtained with the others indicates a similar distribution of subtypes to that described for the corresponding period in France (data not shown) [46,47].

This study has several limitations. Due to its retrospective nature, it includes only virological and epidemiological data. It is not, therefore, possible to assess the clinical impact of respiratory viruses, their nosocomial or community origins or potential associations with bacteria. Another limitation is the absence of international, national or uniform local recommendations concerning the use of mPCR. The local case definition requiring mPCR may have changed, accounting for the gradual increase in the number of tests performed over the study period, from 854 in the first year to 2296 in the last. Thus, no data are available concerning the number of suspected cases not tested by mPCR, although our lower global positivity rate than observed in previous studies may suggest that these tests are widely used by physicians in our hospital group. Concerning the picornavirus group, none of our mPCR test can discriminate between rhinovirus or enterovirus subtypes with a strong robustness. Thus, we cannot assess in the current work any differential distribution or pathogenicity within this large viral group and specific studies are needed. The prevalence of the bocavirus group was clearly underestimated in this study, as these viruses are not detected by the Respifinder 19^®^ or FilmArray^™^ tests (26.4% of the total number of samples tested). However, bocaviruses were found to be very rare in our adult population, accounting for only 1.3% of all viruses identified. A last limitation is the change of mPCR tests used in our laboratory during the study period. All these changes were made with the aim of shortening the time required to obtain the results (48 worked hours for the Respifinder^®^ test, 24 h for Anyplex^™^ and less than 2 h for the FilmArray^®^ test). These changes may have introduced some bias into the viral distribution despite the good year-to-year reproducibility of our results and the local assessment of mPCR performances before the introduction of each test.

Many aspects of the burden of respiratory viruses in hospital settings remain to be investigated. In pediatric populations, in which respiratory virus pathogenicity has been well demonstrated for some viral groups, rapid mPCR assays have been shown to improve the detection of respiratory viruses and to decrease the time required to obtain results, thereby making it possible to decrease the duration of antibiotic treatment or the time spent in the emergency department [25,48]. In adults, evidence for the pathogenicity of respiratory viruses is increasingly evidenced [6,19–23], but antibiotic treatment is often continued after positive PCR tests for viruses, even with a normal chest radiography; further studies and new recommendations are therefore required today [49]. A recent study, paving the way for a future large trial, reported a halving of the duration of antibiotic treatment with a diagnostic strategy based on both PCT and mPCR testing [31]. Respiratory viruses also present a burden in hospital settings through the risk of nosocomial infections. Only influenza tests are currently performed, during epidemic periods, to isolate infected patients and to use specific treatments. Recommendations for patient isolation in cases of infection with other respiratory viruses are unclear, despite recent studies showing that viral infections are neither frequent nor uncommon in patients with severe hospital-acquired pneumonia [22,23]. Larger studies are required to determine the need to enlarge viral detection and patient isolation measures to non-influenza viruses in adults and children.

In conclusion, despite increasing acknowledgement of the pathogenicity of respiratory viruses, extensive assessments of these viruses are still rare in clinical practice because of (i) the high cost of mPCR tests, (ii) the absence of specific treatment or vaccination, except for influenza, and (iii) the reluctance to stop antibiotic treatments in adults. However, the high rates of positivity obtained in this study in real-life hospital settings highlights the large burden of these infections. This is particularly true for groups of viruses that have been largely neglected, such as picornaviruses, when current research efforts focus principally on the development of vaccines and drugs against influenza and RSV. Large prospective studies, including clinical observations and bacterial investigations, are much needed to assess the pathogenicity of picornaviruses and other non-influenza respiratory viruses more precisely, as well as to evaluate and establish new recommendations for their management in hospital settings.

## Supporting information

S1 Fig Distribution, by year, of the viruses detected, according to their respiratory tract distribution (A-B) and the type of medical unit (C-F). (DOCX)

S2 Fig Distribution of respiratory virus groups across types of medical units and location in the respiratory tract: upper (A) and lower (B) respiratory tract. (DOCX)

S3 Fig Distribution, by age group, of respiratory virus groups present in the upper (A) and lower (B) respiratory tract. (DOCX)
