# Supplementary material for: Prevalence of respiratory viruses among adults, by season, age, respiratory tract region and type of medical unit in Paris, France, from 2011 to 2016
Source: PLoS One. 2017 Jul 14;12(7):e0180888. doi: 10.1371/journal.pone.0180888 (PMC5510824; doi:10.1371/journal.pone.0180888)
Supplement: S1 Fig — (DOCX) [file pone.0180888.s001.docx]

**S1 Fig.** Distribution, by year, of the viruses detected, according to their respiratory tract distribution (A-B) and the type of medical unit (C-F).

**A.** Upper Respiratory Tract **B.** Lower Respiratory Tract

**
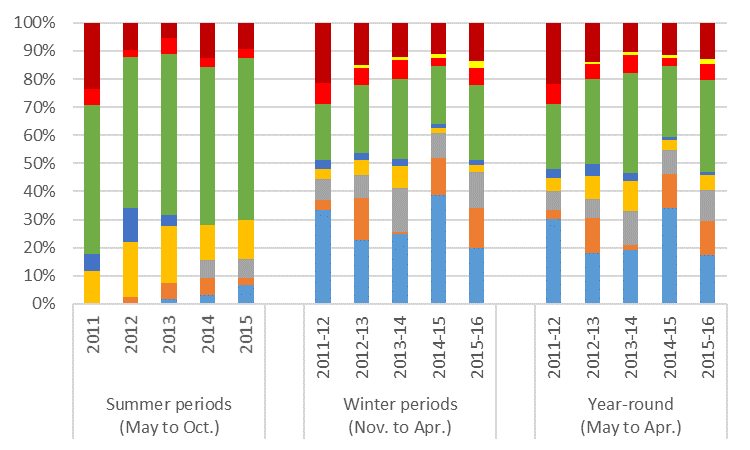

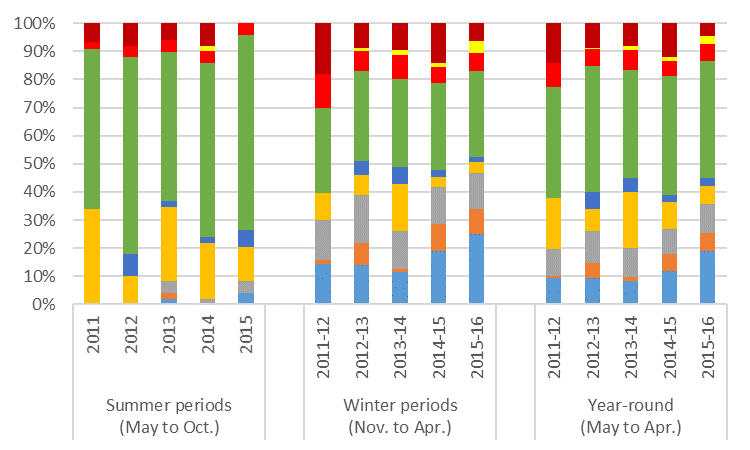
**

**C.** Intensive Care Unit **D.** Medicine Units

**
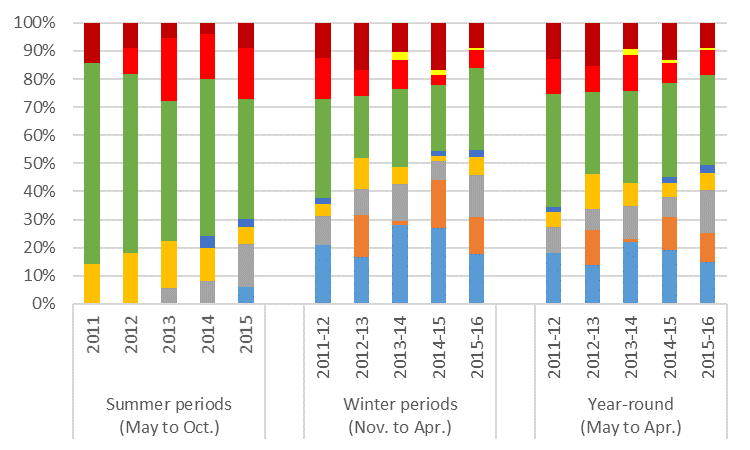

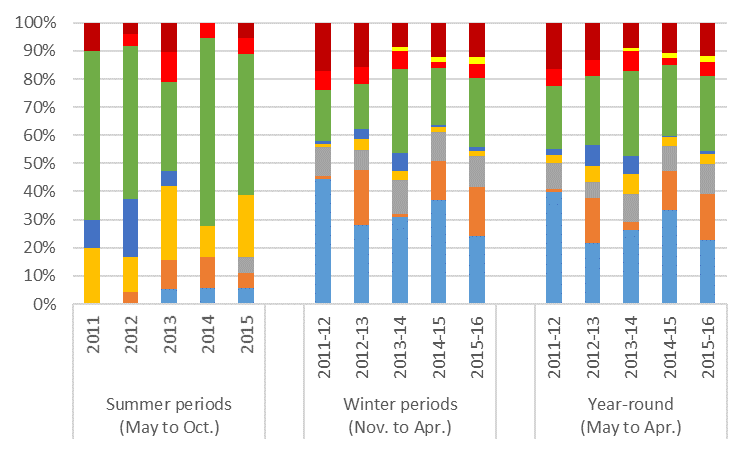
**

**E.** Lung Graft Unit **F.** Pneumology Unit

**
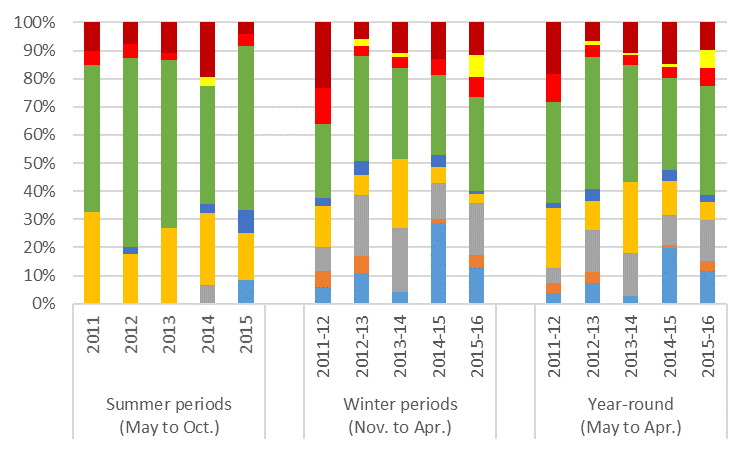

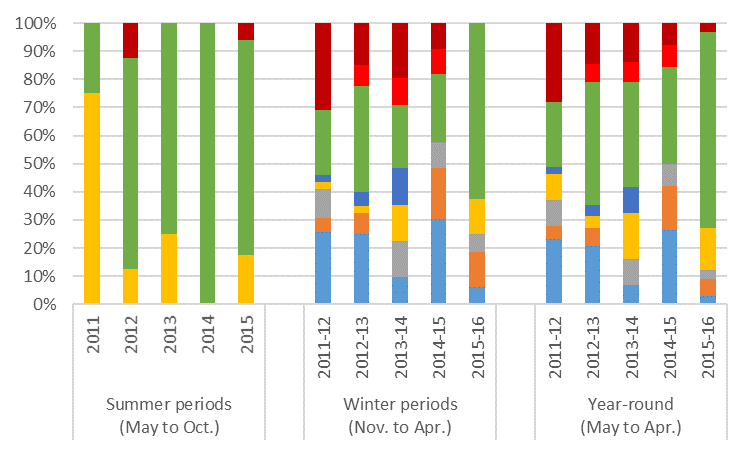
**
