# Supplementary material for: Prevalence of respiratory viruses among adults, by season, age, respiratory tract region and type of medical unit in Paris, France, from 2011 to 2016
Source: PLoS One. 2017 Jul 14;12(7):e0180888. doi: 10.1371/journal.pone.0180888 (PMC5510824; doi:10.1371/journal.pone.0180888)

**S2 Fig.** Distribution of respiratory virus groups across types of medical units and location in the respiratory tract: upper (A) and lower (B) respiratory tract.

**A.**

**
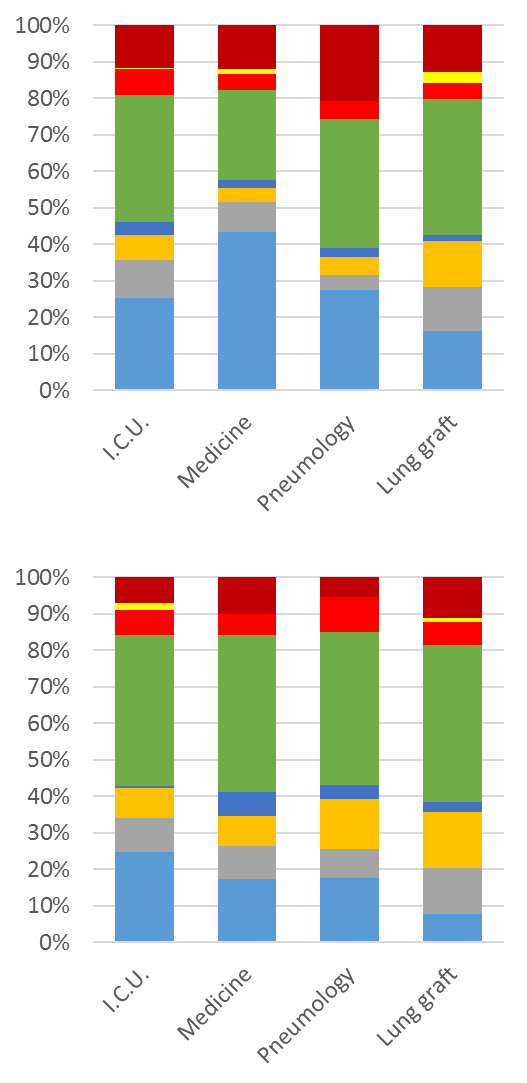
**

**B.**


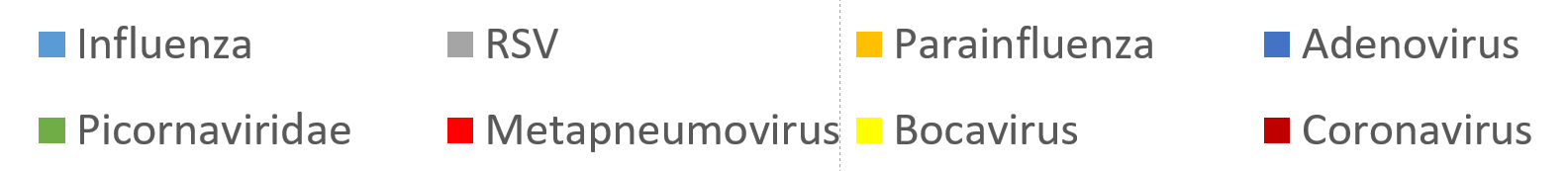

Supplement: S2 Fig — (DOCX) [file pone.0180888.s002.docx]
